# Asexuality vs. sexual interest/arousal disorder: Examining group differences in initial attention to sexual stimuli

**DOI:** 10.1371/journal.pone.0261434

**Published:** 2021-12-16

**Authors:** Julia Bradshaw, Natalie Brown, Alan Kingstone, Lori Brotto

**Affiliations:** 1 Department of Psychology, University of British Columbia, Vancouver, Canada; 2 Department of Obstetrics and Gynaecology, University of British Columbia, Vancouver, Canada; University of Pennsylvania Perelman School of Medicine, UNITED STATES

## Abstract

Attention is considered to be a critical part of the sexual response cycle, and researchers have differentiated between the roles of initial (involuntary) and subsequent (voluntary) attention paid to sexual stimuli as part of the facilitation of sexual arousal. Prior studies using eye-tracking methodologies have shown differing initial attention patterns to erotic stimuli between men and women, as well as between individuals of different sexual orientations. No study has directly compared initial attention to sexual stimuli in asexual individuals, defined by their lack of sexual attraction, to women with Sexual Interest/Arousal Disorder (SIAD), a disorder characterized by a reduced or absent interest in sex coupled with significant personal distress. The current study tested differences in the initial attention patterns of 29 asexual individuals (*M*_age_ = 26.56, *SD* = 4.80) and 25 heterosexual women with SIAD (*M*_age_ = 27.52, *SD* = 4.87), using eye-tracking. Participants were presented with sexual and neutral stimuli, and their initial eye movements and initial fixations to both image types and areas of erotic contact within sexual images were recorded. Mixed-model ANOVAs and *t*-tests were used to compare the two groups on the speed with which their initial fixations occurred, the duration of their initial fixations, and the proportion of initial fixations made to sexual stimuli. On two indices of initial attention, women with SIAD displayed an initial attention preference for sexual stimuli over neutral stimuli compared to asexual participants. This study adds to a growing literature on the distinction between asexuality and SIAD, indicating that differences in early attention may be a feature that differentiates the groups.

## Introduction

### Defining asexuality

Asexuality is defined as a sexual orientation characterized variably as a lack of sexual attraction [1, 2]. Prevalence estimates for asexuality in the general population range from 0.4% to 1% [1, 3, 4]. A more recent study found 3.3% of women and 1.5% of men from a Finnish population had not experienced sexual attraction within the past year [5]. Many asexual individuals report a longstanding disinterest in sex, and for some, identification as asexual occurs after a period of confusion or feeling markedly “different” than allosexual peers (i.e., people who experience sexual attraction) [6]. Other asexual individuals adopt this sexual identity after subsequent education about the asexual label and community.

Research suggests that significant heterogeneity exists within the asexual community [2, 7, 8]. Asexuality exists on a spectrum, and asexual persons use a variety of terms to describe their position on this “ace” spectrum [8]. While approximately two-thirds of ace individuals identify specifically as asexual, just over 10% refer to themselves as “gray-asexual”, experiencing occasional attraction in isolation or with specific partners, to varying levels [8]. Other members of the ace community identify as “demisexual” (approximately 10%), only experiencing sexual attraction when an emotional bond is formed [8]. Asexual individuals also describe a variety of romantic orientations, including but not limited to biromanticism and heteroromanticism [7, 8]. In fact, diversity of romantic experiences within the asexual community has prompted researchers to examine differences between aromantic asexual persons (do not experience romantic attraction to others) and romantic asexual persons, and have found dissimilarities between these groups on variables such as number of romantic and sexual partners, self-reported desire, and personality traits like warmth and nurturance [7]. Additionally, asexual individuals identify as transgender or gender nonbinary at a higher rate (between 12.6% and 25%) than allosexual individuals, indicating considerable gender diversity within the community [7, 8]. The variation observed in the community is not a surprise, as a range of preferences, experiences, and chosen labels also exists amongst other sexual minorities [9].

### Defining Sexual Interest/Arousal Disorder (SIAD)

Sexual Interest/Arousal Disorder (SIAD), a disorder defined by the 5^th^ edition of the *Diagnostic and Statistical Manual of Mental Disorders* (DSM-5), is a sexual dysfunction wherein individuals experience reduced or absent sexual interest and/or arousal, coupled with significant personal distress [10]. Though there is variability in symptom presentation, women must have at least three of the following symptoms for six months to obtain a diagnosis: decreased or absent sexual interest, decreased sexual thoughts or fantasies, a decreased desire to initiate sex or respond to a partner’s initiations, decreased physiological sensations during sexual activity, decreased feelings of sexual pleasure or excitement, and decreased sexual interest/arousal in response to sexual cues [10]. There are two subtypes of SIAD based on symptom duration: (1) lifelong SIAD: symptoms since becoming sexually active; and (2) acquired SIAD: symptoms beginning after a period of satisfactory sexual desire and/or arousal levels [10].

### Differentiating asexuality from SIAD

Some debate has existed in the academic community as to whether asexuality is better understood as a sexual dysfunction [11]. Asexual individuals are sometimes compared to individuals with sexual desire disorders, since both groups display a lack of interest in sexual activity [12]. Despite these commonalities, important factors distinguishing these groups have led researchers to conclude that they are distinct [12]. A key differentiating factor is the presence of significant distress in women with a diagnosis of SIAD. Though asexual individuals may experience distress related to a lack of widespread social acceptance of their asexual identification, they are unlikely to seek treatment to try and “fix” their lack of desire [12]. Conversely, women with SIAD may seek psychological and/or pharmacological treatment to improve their desire levels [13]. As well, asexual individuals are more likely to endorse a consistent, lifelong lack of interest in sex and absence of attraction, whereas most women with a sexual desire disorder, whether in empirical studies or presenting for clinical treatment, report a marked reduction in their sexual arousal as compared to another time in their lives [12, 13]. One study comparing asexual individuals and women with sexual desire disorders found that asexual participants were less likely to be in a relationship, have sexual fantasies, or masturbate, and were more likely to have never participated in sexual intercourse [12]. Importantly, though SIAD symptomology in women can present with some variation [13, 14], in-group diversity is unlikely to be as high as those who identify on the asexual spectrum, by nature of such specific diagnostic criteria. These differences, among others, are supportive of an argument to distinguish between the two groups.

### The role of attention in sexual response

According to Janssen and colleagues’ information processing model of sexual function [15], automatic and voluntary attention play key but distinct roles in the facilitation of sexual response; stimulating an automatic physiological response and a subjective sense of arousal, respectively. Scholars have distinguished between these automatic and controlled processes as having differing impacts on motivations for sex, sexual arousal, and response [15, 16]. Initial (i.e., automatic) attention refers to an unconscious recognition of and engagement with relevant stimuli, prior to any conscious decision to focus on those stimuli [17]. In the context of sexual response, initial attention to sexual stimuli involves an automatic appraisal of a target as sexually relevant and is hypothesized to initiate an automatic genital response [15, 16]. This physiological reaction, according to the cognitive-motivational model, can encourage controlled (deliberate) attention to that stimulus, which, if followed by positive appraisal, can precipitate further physical and subjective arousal, and may lead to engagement in sexual activity [16]. Initial attention, then, may play an important role in the process of sexual response, and may provide crucial insight into group and individual differences in sexual desire.

Eye-tracking can be used to evaluate both initial and controlled attention patterns. Initial attention can be assessed using three indices: time to first fixation, duration of first fixation, and proportion of first fixations to different categories of stimuli. Although there are other parameters of eye-tracking that have been studied (e.g., controlled attention), we have elected to focus on initial attention because it has not been studied before in these groups and because it can reveal an automatic (rather than voluntary) attentional bias.

Prior research indicates that attentional preferences to erotic stimuli differ based on sexual orientation. Dawson and Chivers [18] used eye-tracking technology to study differences in initial attention between gynephilic (attracted to women) men and androphilic (attracted to men) women. They found that while gynephilic men’s initial attention patterns were focused on sexually preferred stimuli (i.e., women), androphilic women’s initial attention patterns were gender-nonspecific. Simply, men were faster to fixate on erotic images of women, their preferred sexual targets, while women did not show a gender-specific preference, exhibited by minimal differences in time to first fixation for erotic images of men and women. Researchers have also found that gynephilic men and ambiphilic (attracted to both men and women) women displayed initial orienting preferences towards sexual images of female targets, fixating more quickly on them relative to male targets [19]. Meanwhile, androphilic women, again, demonstrated gender-nonspecific patterns in their initial attention patterns, fixating similarly quickly to erotic images of male and female targets. This research indicates that sexual orientation seems to impact initial attention patterns towards sexual stimuli. Though these differences in initial attention have been found between different sexual orientation groups [18, 19], no research has compared initial attention patterns of asexual and allosexual individuals, representing a gap in the literature.

Research has revealed that women with sexual difficulties exhibit gaze behavior towards sexual stimuli that differs from their symptom-free counterparts, such that their attention is less easily captured and held by erotic stimuli [20, 21]. An eye-tracking study found that women with arousal/desire difficulties attended less to the genital area of images depicting a man and woman engaging in vaginal intercourse, relative to a group of asymptomatic women, in one measure of initial attention [21]. The sexual dysfunction group displayed shorter initial fixations to the genital area. These findings suggest that initial attention may play a role in women’s sexual concerns. Whether these differences are related to avoidance, distraction, negative associations with erotic cues, or another explanation is unknown.

Presently, no studies have examined initial attention patterns in asexual individuals. Sexual orientation does seem to influence automatic gaze behavior to sexual targets [18, 19] and increased levels of desire tend to correspond with greater attention to erotic stimuli [21–23]. Since asexual individuals generally describe a lifelong lack of desire or interest in sex, have fewer sexual experiences, and, if engaged in a romantic relationship, are less likely to engage in sexual activity within that relationship [2], it follows that for asexual individuals initial visual attention would not be preferentially allocated towards sexual stimuli.

Meanwhile, while women with low desire seem to display less initial attention to erotic areas of imagery than their asymptomatic counterparts [21], research suggests that they are still more likely to have sexual fantasies, a greater number of sexual experiences and partners, and memories of a stronger desire for sex than asexual individuals [12]. It follows, then, that sexual cues may be more salient to them relative to their asexual counterparts, leading to our prediction that women with SIAD would display a greater initial attention preference towards sexual stimuli (vs. non-sexual stimuli), with quicker initial fixations, longer initial fixation durations, and more frequent initial fixations to sexual imagery.

### Current study

Using eye-tracking methodology with a focus on outcomes of time to first fixation, length of first fixation, and number of first fixations to land on sexual images, we hypothesized that SIAD participants would display an initial attention preference for sexual cues, whereas asexual participants would not. More specifically, we predicted that when presented with sexual vs. non-sexual stimuli, heterosexual women with SIAD would be faster to fixate on sexual stimuli, would have longer initial fixations on sexual stimuli, and would make a greater number of first fixations to sexual stimuli over the course of 20 trials than asexual individuals. We also predicted that the group differences in initial attention patterns would be replicated in the context of the erotic areas (i.e., areas of vaginal penetration, cunnilingus, fellatio, etc.) of sexual images: we anticipated that women with SIAD would be faster to fixate and have longer initial fixations on these erotic areas of sexual images compared to the asexual group.

## Materials and methods

### Participants

The data used here were drawn from an existing dataset of eye-tracking variables in samples of asexual persons and women with either lifelong or acquired SIAD [24].

The initial sample consisted of 25 heterosexual women with SIAD and 42 asexual individuals. Heterosexual women were required to meet criteria for a diagnosis of SIAD as per the DSM-5 [10], and this group was comprised of 9 women with lifelong symptoms and 16 women with acquired symptoms. Inclusion criteria were: over 19 years of age, identified as either asexual or heterosexual, had normal color vision or corrected vision, and were fluent in English. Individuals who identified as demisexual were excluded from analyses, since recent surveys have shown that demisexual individuals respond quite differently to asexual individuals on a variety of sex-related measures, including those assessing their attitudes towards sex [8]. Of the original asexual participants, 10 were tested in another city with a different eye-tracking device, so they were eliminated from this study to ensure consistency of measurement. Due to technical issues with the eye-tracker, three additional asexual participants were excluded from eye-tracking analyses only (for a total of 29 asexual participants).

### Procedure

Participants were recruited via online advertising, social media, tear-off ads posted at UBC, ads posted in public locations around Vancouver, British Columbia, and email invitations sent to prior study participants. The research team consulted with an asexual community advisory group for input on recruitment throughout the study. Assessments took place on an ongoing basis from March 2019 –March 2020. A Master’s student in clinical psychology with two years of diagnostic clinical interview experience conducted short phone interviews with interested individuals to clarify study procedures and to screen for eligibility requirements. The pre-session phone interview included questions about sexual orientation, a series of questions to screen for major mental health disorders, and an assessment to evaluate if participants met criteria for a SIAD diagnosis. This diagnosis required a woman to have had an absence or reduction of: interest in sexual activity, sexual fantasies and thoughts, responsiveness to initiation of sex by a partner or desire to initiate sex themselves, excitement or pleasure during sexual activity, arousal in response to sexual cues, and genital or non-genital sensations during sexual encounters. If women reported that they had experienced at least three of these symptoms over a six-month period and experienced clinically significant distress, they were assigned to the SIAD group.

After the interview, consent forms were emailed to participants who fit the inclusion criteria, and interested participants scheduled their in-person sessions to take place at the Sexual Health Laboratory of the senior author. They were then emailed online questionnaires to complete at home using Qualtrics, an online survey software tool for data collection.

Lab appointments took approximately one hour to complete. The participants were informed of the study procedures, and then provided written consent. They were instructed to sit in a chair and complete an implicit association task (data presented in [24]).

Upon completion of the first task, the study coordinator re-entered the room to set up the eye-tracking device for the visual attention task. In order to calibrate the eye-tracking device, participants were instructed to follow a moving circle on a video screen with their eyes until the device was synced with their eye movements. Following calibration and equipment set-up, the study coordinator briefly described the task to the participant and then left the room again. Participants were able to communicate with the study coordinator throughout the session via intercom if they had questions and to inform the coordinator upon completion of a task.

Once the task sequence was initiated, participants were presented with 20 experimental trials, each of which contained a pair of images presented side-by-side on a computer screen for 10 seconds. Participants were instructed to look at the stimuli as they naturally would. For each pair of images, one was sexually explicit and portrayed a nude male and nude female engaging in sexual activity, while the other was a non-erotic image of a clothed man and woman participating in a non-sexual, non-romantic activity. Image pairs were matched for brightness, color, contrast, and size. At the beginning of each trial, a fixation cross was displayed on the screen. Once participants had fixated on the cross, the images were presented. After the presentation of each image pair, participants were asked two questions: “How attracted were you to the sexual image?” and “How attracted were you to the neutral image?” Participants used a computer mouse to report their sexual attraction to each image on a scale ranging from 0 (*not at all sexually attracted*) to 9 (*very sexually attracted*). After the 20^th^ trial, the participant asked the study coordinator to return to the room via intercom and began preparation for a memory task (data presented in [24]).

Upon completion of testing, the study coordinator explained the study purpose to participants and presented a debriefing form. Participants were also given $25 as compensation for their time. The original study from which the data was drawn was approved by the Behavioural Research Ethics Board at the University of British Columbia, #H18-03236, and the Vancouver Coastal Health Hospital Research Ethics Board. The hypotheses and data analytic plan for the original study, which differ from the present investigation, were pre-registered using the Open Science Framework (https://doi.org/10.17605/OSF.IO/ZYS86). Data for the current study are stored on the Open Science Framework (https://osf.io/j23he).

## Measures

### Initial attention

The SensoMotoric Instruments (SMI) RED 500 desktop eye-tracking equipment was used in combination with the SMI Experiment Suite software program to measure participants’ eye movements at the Vancouver site. The SMI is a screen-based, remote sensor eye-tracker that is contact-free; it measures eye movements without requiring contact with the participant. The device uses infrared illumination to track eye movements and gaze position, through bright and dark pupil systems. Once the SMI is successfully calibrated (this study used a 9-point calibration), the pupil movement is converted into gaze data. The eye-tracking device in this study was affixed to a 22-inch, 1920 x 1080 resolution computer monitor. The SMI functioned at a sampling rate of 120 Hz, had gaze position accuracy of 0.4°, and spatial resolution of 0.03°. The SMI does not require the use of a chin rest, as it compensates for small head movements, and can be used with participants who have contact lenses and glasses.

For this study, we assessed initial attention via three indices: time to first fixation (i.e., how quickly the eye lands upon a specific area for the first time, measured in milliseconds), duration of first fixation (i.e., length in milliseconds of the first fixation on an area), and the proportion of first fixations (i.e., which image type participants fixated on first for each trial). We examined initial attention patterns towards sexual vs. non-sexual images, as well as initial attention towards the erotic area (i.e., the specific area of vaginal penetration, fellatio, cunnilingus) of sexual imagery. BeGaze software was used to calculate and determine the entry time (time to first fixation) and first fixation duration variables for our sample. BeGaze defines entry time as “Average duration from start of the trial to the first hit of an area of interest (AOI) in milliseconds.” First fixation duration is defined as “The duration of the first fixation in an AOI (if any) in milliseconds.” Fixations were defined as moments when a saccadic eye movement was not recorded for at least 50 milliseconds (i.e., the eyes were not in motion for at least 50 milliseconds). Saccades were defined based on a peak velocity threshold of 40 degrees/second, with a peak velocity window from 20% to 80% of the saccade length, as per the SMI’s built-in event detection algorithm. If there was a blink at the start or the end of the saccade then the saccade was discarded. Group differences were analyzed, with the assumption that greater initial attention to sexual imagery as compared to neutral imagery indicates an initial attention preference for erotic cues.

### Self-report measures

#### Demographics

We assessed a variety of demographic questions, including age, ethnicity, relationship status, number of previous sexual partners, and frequency of masturbation with eight answer options ranging from “No, I do not masturbate” to “Yes, more than once a day.” A multiple-choice question about sexual orientation was also included, with seven options, and was used to verify group membership.

#### Asexuality

The Asexuality Identification Scale (AIS) was used to assess participants’ asexuality. The AIS is a valid and reliable 12-item self-report questionnaire that includes statements such as “I lack interest in sexual activity”, and requires participants to respond on a Likert-type scale ranging from 1 (*completely false*) to 5 (*completely true*) [25]. The AIS has a total possible score of 60, with higher scores indicating a higher probability of an asexual identity. Typically, 40/60 is the cut-off score to differentiate asexual individuals from allosexual individuals. For our sample, the AIS demonstrated excellent internal consistency (α = 0.95).

#### Sexual desire

The Sexual Desire Inventory-2 (SDI-2) was used to assess both solitary and dyadic desire via 14 items, with a possible total score of 112 [26]. Higher scores indicate higher levels of desire. Dyadic desire items measure a person’s level of interest in engaging in sexual activity with a partner, and include questions such as “When you have sexual thoughts, *how strong* is your desire to engage in sexual behavior with a partner?”, with a Likert-type scale response ranging from 0 (*no desire*) to 8 (*strong desire*). Solitary desire, or the desire to engage in sexual activity with oneself, is measured with items such as “*How important* is it for you to fulfill your desires to behave sexually by yourself?” and similar Likert-type answer choices. The Sexual Desire Inventory, from which the SDI-2 was adapted, has been found to have strong concurrent validity; solitary and dyadic desire scores correlate with frequency of solitary and dyadic sexual behavior, respectively [26]. For our sample, internal consistency on the SDI-2 was good (α = 0.86).

#### Sexual distress

A sexual distress measure was included in order to describe our sample, as distress is considered a differentiating factor between SIAD and asexual groups. The Female Sexual Distress Scale-Revised (FSDS-R) was used to measure sex-related distress [27]. The FSDS-R is a 13-item self-report scale that examines frequency of distress related to sexual activity over the prior 30-day period. Items include questions such as “How often did you feel worried about sex?” with response options ranging from 0 (*never)* to 4 *(always)* [27]. A total score of 15 or higher signifies the presence of sex-related distress [27]. The FSDS-R has high test-retest reliability and discriminant validity; it has shown to be successful at discriminating between women with sexual desire disorders and those without (at 93% accuracy), with significantly higher mean scores being found in women with clinically low desire regardless of recall period (*p* < .001) [28]. The FSDS-R demonstrated excellent internal consistency within our sample (α = 0.97).

#### Depression

A measure of depression was included to describe our sample, and because depression has been previously linked to differences in attention via eye-tracking [29]. Depressive symptoms were measured using the Beck Depression Inventory-II (BDI-II) [30]. The BDI-II is a widely used measure of depression, with 21 items formatted as themed groups of statements. Themes include items such as “Pessimism,” “Loss of Pleasure,” and “Worthlessness” [30]. Responses are statement options of increasing intensity, from 0 (e.g., *I do not feel I am worthless*) to 3 (e.g., *I feel utterly worthless*), and respondents are asked to choose the statement that best matches their mood over the last two weeks. A total score of 15 or higher indicates a high likelihood of depression. The BDI-II has high internal consistency (α = 0.92) [30] and convergent validity; BDI-II scores have been found to positively correlate with the Patient Health Questionnaire-9 (*r* = 0.75), another measure of depression [31]. Internal consistency for this measure was high in our sample (α = 0.91).

#### Sexual aversion

A measure of sexual aversion was included to describe our sample, a measure that has not been studied before in these groups. The Sexual Aversion Scale (SAS) is a 30-item self-report measure that evaluates fear and avoidance of sex and has demonstrated high test-retest reliability [32]. Items on the scale are formatted as statements such as “The thought of sex makes me nervous,” and test-takers decide applicability of statements to themselves on a scale from 1 (*not at all like me*) to 4 (*a lot like me*) [32]. Total scores are used to reflect levels of sexual aversion, with higher scores indicating a greater presence of sexual aversion. The SAS demonstrated high internal consistency in the current sample (α = 0.90).

### Data analysis

#### Power analysis

This study was based on secondary data analysis. For this study, we were sufficiently powered to examine group differences in initial attention, the primary aim of the paper. We anticipated detecting group differences in initial attention measures of medium magnitude. An a priori power analysis conducted using G*Power 3.1 software for mixed-model analyses of variance (ANOVAs) indicated that to achieve a medium effect size (*η*^2^ = 0.06) with power set at .80, we required a total sample size of *N* = 46.

#### Initial visual attention analyses

To test our initial visual attention hypothesis, the eye-tracking software was used to create three regions of interest (ROIs): the sexual image, the non-sexual image, and the erotic area (i.e., the area of vaginal penetration, cunnilingus, fellatio, etc.). Initial attention to the ROIs in this study was assessed through three dependent variables: the time to first fixation for each ROI, duration of the first fixation on the ROIs, and proportion of first fixations to the sexual image. Participants were scored on these dependent variables on each of the 20 trials. Time to first fixation and first fixation duration scores were averaged across all trials, to calculate the mean scores of each dependent variable for each ROI (sexual image, non-sexual image, and erotic area). To calculate the proportion of first fixations to the sexual image, each participant’s number of first fixations to this ROI was divided by their total number (20) of first fixations. Of note, slower times to first fixation on a stimulus translate as higher scores but indicate *less* of an attentional preference for said stimulus. In contrast, greater duration of first fixations and greater proportion of first fixations indicate more of an attentional preference.

To analyze our first hypothesis that women with SIAD would display a greater initial attention preference to sexual stimuli vs. neutral stimuli compared to asexual participants, we first conducted two separate 2 (group: asexual vs. SIAD) x 2 (image type: sexual vs. neutral) mixed-model analyses of variance (ANOVAs) for two of the dependent variables (i.e. time to first fixation, duration of first fixation). These analyses allowed us to examine interactions between group and image type on these initial attention measures. We also conducted an independent samples *t*-test to examine the proportion of first fixations on sexual stimuli by group. To investigate potential group differences in attention to the erotic areas within sexual images, we conducted two independent samples *t*-tests for two of the dependent variables: time to first fixation and duration of first fixation.

## Results

### Sample characteristics

Table 1 lists the demographic information of the participants, organized by group (i.e., asexual, SIAD). The two groups were similar on many demographic variables, including age, gender identity, ethnicity (the majority identified as White/Caucasian), level of education (most attended some amount of college or obtained a college degree), and sexual assault history. Several asexual individuals (17.2%) reported transgender experience (i.e., identifying with a gender that was not assigned to them at birth), compared to women with SIAD, all of whom identified as cisgender. A chi-squared test indicated that women with SIAD were more likely to identify as romantic than aromantic, compared to asexual individuals, *χ* ^2^ (1, *N* = 56) = 7.53, *p* = .006. Similarly, women with SIAD were more likely to report themselves as dating (44%) or married/common-law (44%) than their asexual counterparts, who were more likely to report themselves as single (80.6%), *χ*^2^ (2, *N* = 56) = 26.44, *p* < .001. An independent samples *t*-test revealed that asexual individuals scored higher on the Asexuality Identification Scale [25] than women with SIAD, *t*(55) = 10.94, *p* < .001. Regarding income, there was a difference of group, with a greater percentage of women with SIAD (65.2%) reporting an annual income of >$60,000 relative to asexual individuals (29.2%), *χ* ^2^ (7, *N* = 47) = 7.09, *p* = .029.

**Table 1:** Demographic information of participants by group.

Variable	Asexual (*n* = 32)	SIAD (*n* = 25)
Age *M (SD)*	26.56 (4.80)	27.52 (4.87)
Gender identity (%)		
Woman	89.7	100
Non-binary	10.3	0
Trans-experience (%)*	17.2	0
Romantic Orientation (%)*		
Aromantic	25.8	0
Romantic	74.2	100
Relationship status (%)*		
Single	80.6	12
Dating	12.9	44
Married/common-law	6.5	44
Current relationship length in yrs. *M* (*SD*)	3.57 (4.32)	4.59 (2.87)
AIS *M* (*SD*)*	48.74 (6.40)	27.36 (8.36)
Sexual assault history (%)	50	41.7
Ethnicity (%)		
East Asian	15.6	28
South Asian	0	8
Southeast Asian	3.1	0
First Nation	0	4
Hispanic	3.1	8
Middle Eastern	0	8
White/Caucasian	62.5	44
Other	15.6	4
Level of education (%)		
High school	6.3	0
Attended some college	28.1	20
College degree	50	52
Post-graduate degree	15.6	28
Income Category (annual)*		
<$20,000	16.7	21.7
$20,000 - $39,999	20.8	4.3
$40,000 - $59,999	33.3	8.7
$60,000 - $79,999	16.7	4.3
$80,000 - $99,999	4.2	21.7
$100,000 - $119,999	4.2	13.0
$120,000 - $139,999	0	0
$140,000 - $159,999	4.2	17.4
>$160,000	0	8.7

AIS, Asexuality Identification Score.

*Group differences between asexual and SIAD, *p* < .05

Group comparisons on sexual desire, sex-related distress, depressive symptoms, sexual aversion, and sexual activity frequencies are presented in Table 2.

**Table 2:** Sex and mental health questionnaire scores, by group.

	Asexual (*n* = 32)		SIAD (*n* = 25)		
Variable	*M*	*SD*	*M*	*SD*	*d*
SDI-2 Total Score*	16.72	12.17	32.86	14.92	1.19
SDI-2 Dyadic Score*	5.87	7.60	23.8	9.72	2.05
SDI-2 Solitary Score	10.84	8.25	9.00	6.43	0.25
FSDS-R*	8.47	10.37	30.16	12.67	1.87
BDI-II	11.07	10.88	13.04	10.30	0.19
SAS*	56.68	12.97	69.16	15.55	0.87
Frequency of Masturbation*	3.17	1.76	2.36	1.15	0.54
Number of Sexual Partners	6.32	7.72	8.32	10.22	0.22

SDI-2, Sexual Desire Inventory-2; FSDS-R, Female Sexual Distress Scale-Revised; BDI-II, Beck Depression Inventory-II; SAS, Sexual Aversion Scale. Independent *t*-tests were used to examine group differences.

### Time to first fixation

The 2 (group: asexual vs. SIAD) x 2 (image: sexual vs. neutral) mixed model ANOVA revealed a significant two-way interaction, *F*(1,52) = 11.23, *p* = .002, η_p_^2^ = 0.18, suggesting that women with SIAD were significantly faster to first fixate on sexual stimuli than neutral stimuli relative to asexual participants. Follow-up independent samples *t*-tests revealed significant group differences for the time to first fixation on the sexual image, *t*(52) = 2.16, *p* = .035, *d* = 0.60, with SIAD participants fixating significantly faster than asexual participants on the sexual image. Significant group differences were also found for the time to first fixation on the neutral image, *t*(52) = 3.35, *p* = .002, *d* = 0.91, with asexual participants fixating on the neutral image faster than the SIAD group. An independent samples *t*-test revealed no significant effect of group on time to first fixation to the erotic area ROI, *t*(52) **=** 1.55, *p* = .127, *d* = 0.42. Table 3 shows the group means and standard deviations for the time to first fixation on the sexual image, neutral image, and erotic area.

**Table 3:** Time to first fixation by group.

	Asexual (*n* = 29)			SIAD (*n* = 25)		
Region of Interest	*M* (ms)	*SD* (ms)	95% CI	*M* (ms)	*SD* (ms)	95% CI
Sexual	1111.64	518.46	939.50, 1283.78	838.95	385.73	653.55, 1024.35
Neutral	1410.51	709.03	1137.05, 1683.98	2080.75	761.84	1786.22, 2375.28
Erotic Area	2123.55	977.14	1751.87, 2495.24	1729.19	878.51	1683.00, 2198.95

### First fixation duration

The 2 (group: asexual vs. SIAD) x 2 (image: sexual vs. neutral) mixed model ANOVA revealed no statistically significant two-way interaction, *F*(1,52) = 0.04, *p* = .838, η_p_^2^ = 0.00. There was no main effect of stimulus type (sexual vs. neutral), *F*(1,52) = 0.25, *p* = .618, η_p_^2^ = 0.01, and no main effect of group, *F*(1,52) = 1.08, *p* = .303, η_p_^2^ = 0.02, suggesting that asexual individuals and women with SIAD had similar first fixation durations to sexual and neutral images. An independent samples *t*-test revealed no significant group differences on first fixation duration to the erotic area ROI, *t*(52) = 1.40 *p* = .166, *d* = 0.39, suggesting that women with SIAD did not exhibit longer initial fixations to the erotic area than asexual individuals. Table 4 displays the group means and standard deviations for first fixation durations on the sexual image, neutral image, and erotic area.

**Table 4:** First fixation duration by group.

	Asexual (*n* = 29)			SIAD (*n* = 25)		
Region of Interest	*M* (ms)	*SD* (ms)	95% CI	*M* (ms)	*SD* (ms)	95% CI
Erotic	320.60	65.56	295.53, 345.67	308.68	69.25	281.68, 335.69
Neutral	328.70	54.29	305.01, 352.39	312.07	72.91	286.56, 337.58
Erotic Area	500.55	170.33	435.76, 565.34	556.68	112.58	510.21, 603.15

### Proportion of first fixations to sexual stimuli

The independent samples *t*-test revealed a significant difference between the two groups, *t*(52) = 2.20, *p =* .032, *d* = 0.61, suggesting that across the 20 experimental trials women with SIAD were more likely to fixate first on sexual stimuli compared to the asexual group. Table 5 shows the group means and standard deviations of the proportion of first fixations to the sexual stimuli.

**Table 5:** Proportion of first fixations to erotic stimuli by group.

	Asexual (*n* = 29)			SIAD (*n* = 25)		
	*M*	*SD*	95% CI	*M*	*SD*	95% CI
First Fixation Sexual Ratio	0.57	0.13	0.52, 0.62	0.65	0.14	0.59, 0.70

## Discussion

The aim of this study was to investigate patterns of early visual attention to sexual stimuli among a sample of asexual persons and women with SIAD. On two indices of initial attention, we observed group differences in gaze behavior towards sexual imagery in the predicted direction, thus partially supporting our hypothesis. Specifically, women with SIAD were quicker to first fixate on sexual imagery (as compared to the neutral imagery) than the asexual individuals, and they were more likely to first fixate on the sexual imagery relative to the asexual group. In contrast to our predictions, group differences did not emerge in first fixation duration on sexual vs. neutral images, nor on the first fixation duration to the erotic area. Further, our hypothesis that women with SIAD would be significantly faster to fixate on the erotic area than the asexual participants was not supported by the data.

In the context of sexual vs. neutral stimuli, although both groups were faster to fixate on the sexual image than its neutral counterpart, this effect was greater for women with SIAD than asexual participants. The fact that both groups’ early attention was more quickly drawn to the sexual image may be related to social information, since initial fixations may be driven by a built-in mechanism to extract socially relevant information from a complex scene [33]. For example, participants preferentially attend to the eyes of people in an image before glancing at other body parts or background features, likely due to the social cues that can be extrapolated from them [33]. Given that babies begin to preferentially glance at eyes by about two months of age [34], it seems that there may be an innate tendency to attend to socially relevant information first. In our study, participants were presented with two images featuring social interactions, but the sexual scene was arguably more socially complex, involving touching and physical intimacy. This complexity could explain our findings that both groups, asexual and SIAD, displayed a tendency to fixate faster on the sexual image than the neutral one. Sexual cues are likely to be *more* socially relevant for women with SIAD than for asexual individuals, however, as women with SIAD likely remember a time when they felt a greater desire for sex. These factors may account for their quicker orienting to the erotic image compared to the asexual group. Though we did not find a significant difference between groups on the time to first fixation for the erotic area, both groups took twice the amount of time to orient to this area compared to the erotic image as a whole. This slower orienting may be due to the tendency of humans to attend to the eye area of faces before other components of social scenes, to gather social information [33].

While we found that asexual individuals did look 2.5% longer at the neutral than the sexual image, this trend did not reach statistical significance (i.e., they did not show a bias towards either image type), and there were minimal differences between groups when examining first fixation durations to sexual and neutral stimuli. The lack of effect of image type on fixation duration for both groups could be accounted for by an automatic tendency to gather information from both images, assessing the entire stimulus display prior to deliberately shifting attention to one image [35, 36]. Participants may have been faster to orient to the sexual image due to its social complexity, but once their attention systems identified the image as sexual, they may have moved on to process the imagery on the rest of the screen in order to understand the scene in its context. Similarly, for the first fixation duration to the erotic area, group differences were not significant. Given that these first fixations on the erotic area happened later on compared to the fixations on the sexual and neutral stimuli generally, these fixations may be more indicative of voluntary attention than purely automatic attention.

Proportionally, women with SIAD were significantly more likely than the asexual individuals to have first fixations in each trial which landed on the sexual stimuli, rather than the neutral stimuli. This finding indicates an initial attention bias towards sexual imagery for women with SIAD, and an absence of a strong initial attention bias for asexual participants. Put simply, this result lends support to the idea that women with low desire are more likely to have a built-in preference for erotic cues, while asexual persons may have an intrinsic disinterest towards these cues.

Our findings partially support the idea that asexual individuals do not display an initial attention preference towards sexual imagery, whereas women with SIAD do. In fact, on one of our measures (duration of first fixation), the asexual group demonstrated a tendency to fixate on neutral imagery for around 2.5% longer (not significant) than sexual imagery. Asexual participants also displayed less of a tendency to fixate first on the sexual stimuli over the neutral stimuli, relative to our SIAD group. The lack of initial attention preference for sexual stimuli in the asexual group supports previous research findings that link sexual orientation to initial attention preferences [18, 19]. Since asexual individuals are characterized by their lack of sexual attraction to others, it makes sense that they do not have a strong automatic visual preference for sexual stimuli. Meanwhile, on two of our initial attention measures, the heterosexual women with SIAD displayed an initial attention bias for sexual imagery over neutral imagery.

Our results complement the findings of Brown and colleagues [24], who observed that women with SIAD and asexual individuals exhibited different patterns of controlled attention to sexual cues. Women with SIAD displayed a controlled attention preference for erotic images and areas of sexual contact, while asexual individuals did not [24]. The initial attention bias shown by our sample of women with SIAD towards sexual stimuli is in line with their controlled attention to these stimuli. This suggests that though women with SIAD may display less attention to sexual images than asymptomatic women [21], they still demonstrate an automatic *and* voluntary visual bias to those images. It is also worth noting that the initial attention patterns of asexual individuals were similar to their patterns of controlled attention; on the majority of measures they showed no preference for sexual stimuli.

### Situating the findings within a model of attention

The incentive motivation model (IMM) may be useful to further consider our findings. The IMM was originally used to describe a broader theory of motivation for sexual motivation, arousal, and behavior [37, 38]. Laan and Both [39] have argued that sexual desire is a consequence of both an innate responsive sexual desire system that draws an individual’s attention towards sexual stimuli, and the sexual stimuli in an environment that activate such a response. Given our findings that asexual individuals demonstrated less of an initial attention bias towards sexual stimuli, it is possible that their lack of attention to these stimuli may be a cause of their lack of attraction, among other factors. Alternatively, it is possible that asexual individuals’ lack of initial attention to sexual stimuli is instead a *consequence* of their lack of attraction. If asexual individuals have an intrinsically reduced or absent desire for sexual activity, sexual stimuli would be unlikely to trigger an incentivized response, and thus would fail to capture automatic attention. If no strong, innate sexual desire system exists within an asexual person, according to the IMM erotic stimuli may be less attentionally relevant to this group than other types of stimuli. Future research should further examine attention patterns among asexual persons representing the entire spectrum of ace in order to elucidate how well the IMM accounts for their experiences of attraction or lack thereof.

### Clinical implications

Given previous data demonstrating that women with sexual dysfunction show *reduced* initial attention to sexual imagery compared to asymptomatic women [21], this might be important for formulating treatment goals among women with SIAD seeking clinical assistance. One experimental study found that women with low desire who completed a mindfulness task involving a focus of attention on the sensations and feelings in their bodies and their genitals while viewing an erotic film experienced heightened subjective arousal and genital blood flow [40]. Multi-session mindfulness-based cognitive therapy (MBCT) has also been shown to be effective for women with SIAD [41]. Clinicians treating women diagnosed with SIAD may even find it beneficial to assess visual attention to erotic stimuli prior to and following treatment, which may help establish whether treatment gains are mediated by improvements in early and sustained attention to sexual cues. Currently, no studies have used eye-tracking to examine the impact of greater attention to sexual stimuli on desire/arousal concerns in women. Future research should determine whether an association exists between increased attention to sexual stimuli and improved sexual arousal/desire. It will also be important for researchers to determine which types of attention (i.e., controlled, initial, or both) are associated with a reduction in symptoms for women with SIAD.

The group differences we have found further bolster the conclusion that asexuality and SIAD are distinct, and highlight the need for clinicians to exercise caution when diagnosing SIAD to ensure that a careful differential diagnosis of asexuality is ruled out. An asexual person may suffer distress associated with having an asexual identity in a sex-focused culture, or with trying to satisfy a romantic partner, but that distress should be differentiated from that experienced by women meeting criteria for SIAD. Clinicians should also be knowledgeable about other identities on the asexual spectrum, such as demisexual and gray-asexual, to avoid a diagnosis of SIAD when an ace spectrum identity may be more fitting for the patient. When presented with a patient who is unsure about the source of their sex-related distress or about their sexual identity, clinicians should be careful to conduct a thorough assessment before assuming the individual has a sexual desire disorder. Assessment may include not only standard diagnostic questions related to SIAD, but also the AIS [25] to assess for asexual characteristics and differentiate from SIAD [12]. Of note, the AIS should be used to frame questions pertaining to asexuality, as it may not be as reliable for classifying those with identities other than asexual on the ace spectrum [8]. For clients who are uncertain about their sexual identity, clinicians may also want to recommend further reading on asexuality as a sexual orientation, particularly via the AVEN website, and to provide curious individuals with access to local meet-up groups in their area.

### Limitations and future directions

To our knowledge, this was the first study to compare the initial attention patterns of asexual individuals and women with SIAD to sexual stimuli, and further studies of a similar nature are needed to make more definitive interpretations of our findings. Our sample size was limited due to Covid-19 related interruptions in in-person testing, though we were sufficiently powered to find the predicted effects. We also did not include a group of sexually active individuals without a sexual desire disorder, and we recognize that the absence of a control group may limit our ability to draw firm conclusions regarding attention biases in our groups.

There are a few key differences between our groups that may affect their initial attention, and a lack of prior research to speak to them. For example, our findings may be affected by the time course of our participants’ low desire. We collapsed acquired and lifelong SIAD participants (normally distinguished by symptom onset) into one group in order to maximize power. We also only included asexual participants who endorsed lifelong asexuality, and thus did not take into account asexual folks who report allosexuality at an earlier point in their lives. We suggest that future studies with larger samples examine initial attention with groups separated by the length of time they have experienced low sexual attraction.

Our groups also differed on relationship status, as the majority of our SIAD group were in relationships while most asexual folks were single. While it is possible that the emotional impact of having low attraction while in a romantic relationship could affect initial visual attention, our SIAD sample was not large enough to separate by relationship status while maintaining sufficient power. Thus, we encourage future researchers to examine this variable further.

Similarly, we acknowledge that our asexual group included a span of romantic attractions outside of heteroromantic, including some aromantic individuals, unlike our SIAD group. Given that our sexual images were heteronormative in nature (i.e., depicted men and women engaging in sexual acts), this may have influenced our results. While we are not aware of any studies that link romantic orientation to early attention to sexual stimuli, future research could explore the relationship between initial attention to sexual stimuli depicting different dyad types (e.g., same-sex, opposite-sex, transgender folks) and romantic orientation amongst asexual individuals.

Our groups differed on a measure of sexual aversion, with SIAD women reporting more fear and avoidance of sex than asexual individuals. While there is evidence of a relationship between specific phobias and initial orienting to threatening stimuli, prior research has largely found that phobic participants’ quicker initial orienting to these stimuli is followed by less controlled attention, which some authors have interpreted as visual avoidance [42–44]. Since Brown et al. found that women with SIAD displayed greater controlled (voluntary) attention to sexual stimuli than an asexual group [24], we have not attributed our findings to differences in sexual aversion. We suggest that future studies examine initial attention to sexual stimuli in participants of the same sexual orientation who differ on levels of sexual aversion.

We recruited asexual folks who adopted a rather strict definition of a lack of sexual attraction towards others, and we did not recruit aces who primarily identified as demisexual or gray-asexual; thus, our findings cannot be generalized to these subpopulations. Acknowledging that asexuality exists on a spectrum [8], future research in this area should include participants who identify as demisexual. Comparing the initial and controlled attention patterns of individuals across the ace spectrum may provide more insight into the ways in which they differ. Future studies could also compare the attention patterns of demisexual individuals with women with both SIAD subtypes to examine if the group differences in attention biases found in this study are replicated across these groups.

It is conceivable, although to our knowledge, untested, that prior sexual experiences could influence a person’s initial attention to sexual cues, as those cues may have personal relevance. In this study, women with SIAD and asexual individuals reported similar numbers of past sexual partners, despite our findings that women with SIAD display some initial attention bias towards erotic cues while asexual individuals do not. However, a qualitative study of asexual individuals by Hille and colleagues [45] revealed that asexual individuals across the spectrum are more likely to define “sex” as any partnered activity involving the genitals or anus, as opposed to heterosexual persons, who are more likely to define sex by penetrative activities [46, 47]. The majority of our sexual images involved penile-vaginal penetration, which may be the desired sexual activity type for SIAD women. Additionally, past number of sexual partners is an indirect measure of past sexual experience, and a more comprehensive measure of participants’ erotic experiences should be used for future studies of this nature.

The sexual images used for this study showed several types of sexual activity, the majority of which were penetrative sex, which may have affected our initial attention findings. It is possible that women with SIAD may be more likely to automatically attend to penetrative sex than oral sex, which could have skewed our results. We did not have enough images overall to carry out an analysis of sexual activity type to investigate this issue further.

A meta-analysis conducted on the utility of implicit measures in sex research stressed the need for further eye-tracking research in the field, particularly as it pertains to sexual dysfunction [48]. Specifically, the authors highlighted measures of initial attention as a critical focus of future research, to ascertain differences between automatic and voluntary attention patterns in clinical samples, and to comprehend where in the attentional process an impairment first occurs. Greater knowledge of attentional biases will be necessary to further understand the cognitive aspects of sexual dysfunction, and to develop appropriate treatment plans for individuals who seek help [21, 48].

## Conclusion

To our knowledge, this study is the first to examine the initial attention patterns of asexual individuals and women with SIAD towards sexual cues. On two indices of initial attention, we found that women with SIAD had an initial attention preference towards sexual imagery over neutral imagery, compared to asexual individuals who did not show a preference. This new finding adds to prior research linking sexual orientation to initial attention patterns and adds to the growing collection of studies that distinguish asexuality from sexual desire disorders. This study also accentuates the need for clinicians to be thorough in their assessments of individuals with an absence of sexual interest/arousal, to ensure that a diagnosis of SIAD is not made when a patient may instead be on the ace spectrum. Additionally, clinicians may want to consider mindfulness-based tasks as a component of treatment for clients with SIAD, in order to encourage their attention towards sexual cues and sensations in their bodies. Future studies should examine the association between improved attention to sexual cues and changes in sexual arousal/interest.
